# Letter on: 3D figure of epilepsy syndromes

**DOI:** 10.1002/epi4.12762

**Published:** 2023-05-19

**Authors:** Douglas R. Nordli, Se Hee Kim, Christian M. Korff, Mohamed Taha, Andrew Kim, Chalongchai Phitsanuwong, Douglas R. Nordli

**Affiliations:** ^1^ Mayo Clinic Florida Jacksonville Florida USA; ^2^ Severance Hospital Seoul South Korea; ^3^ University Hospitals, Childrens Hospital Geneva Switzerland; ^4^ University of Chicago Chicago Illinois USA; ^5^ Pediatrix TeleNeurology Program Sunrise Florida USA

Dear Editor,

We read the article by Nabbout at all, showing a three‐dimensional figure of epilepsy syndromes with great interest.^1^ We were struck by the elegance of the figure and its pedagogic utility. For more than a decade, we have been using a similar pie‐shaped figure that we believe complements the one published by Nabbout and colleagues.^2^ Our figure categorizes the common EEG findings found in people with epilepsy so that the EEG results can be easily incorporated into the clinical formulation of the epilepsy syndrome. This educational tool has been used in multiple centers in the US, Europe, and Asia. We believe it fills the gap between seizure semiology and the ultimate diagnosis of an epilepsy syndrome.

Figure 1 shows the tool is in its simplest form. There are six quadrants. In the top or northern portion the EEG background is normal, while in the southern hemisphere, the EEG background shows slowing, whether diffuse or focal. In the western or left side of the diagram the abnormalities are diffuse or multifocal. In the eastern hemisphere the EEG shows focal abnormalities. In Category 1 epileptiform discharges are rare, and can be either diffuse or focal. In Category 4b the EEG is often diffusely abnormal and has discontinuity, electrodecrements or both. Sometimes, the EEGs may show striking asymmetries between the hemispheres which may indicate an underlying focal abnormality. An updated figure is provided with the latest naming as of 2022 (Figure 2).

**FIGURE 1:**
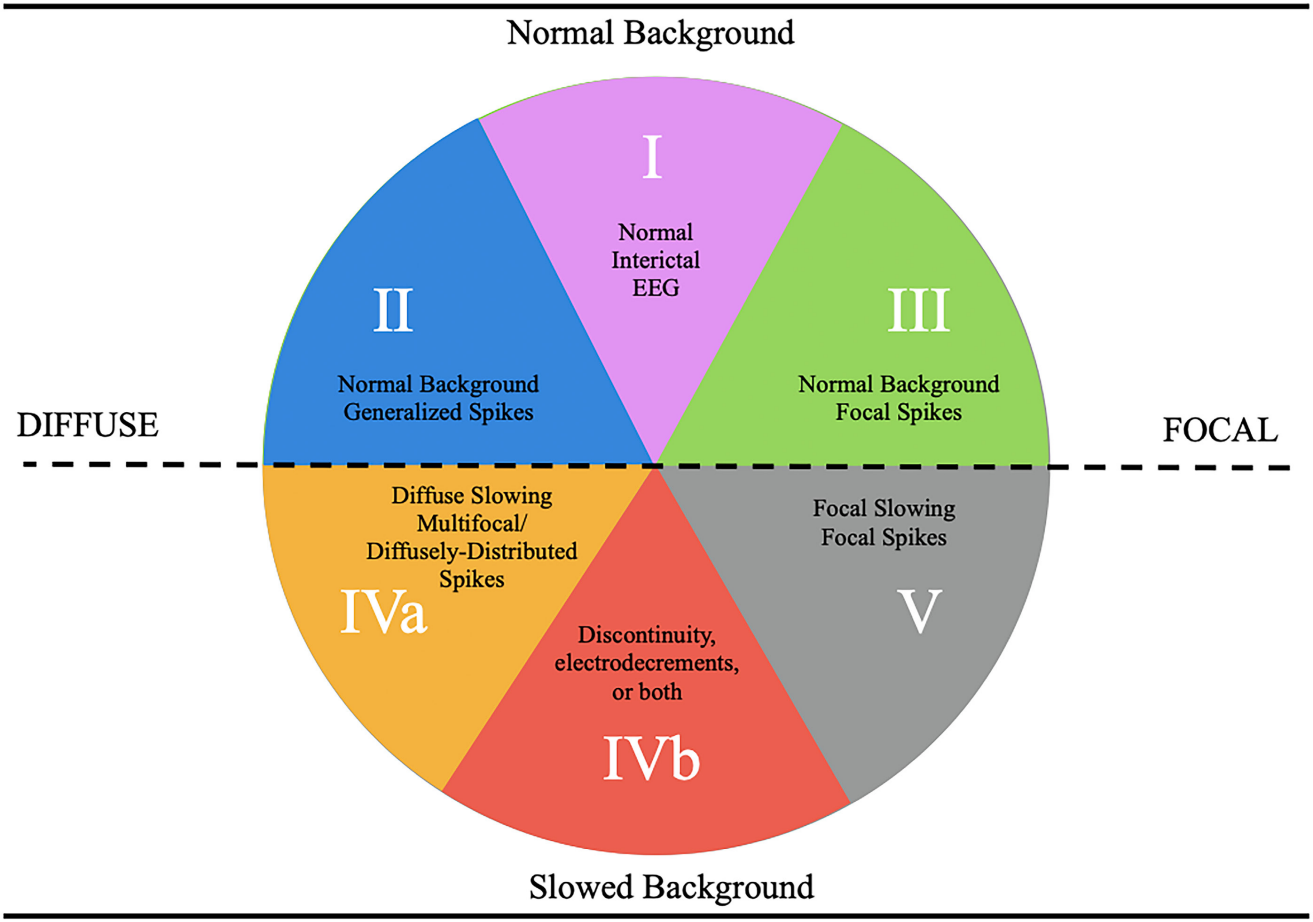
Five categories of EEG findings in the epilepsies.

**FIGURE 2:**
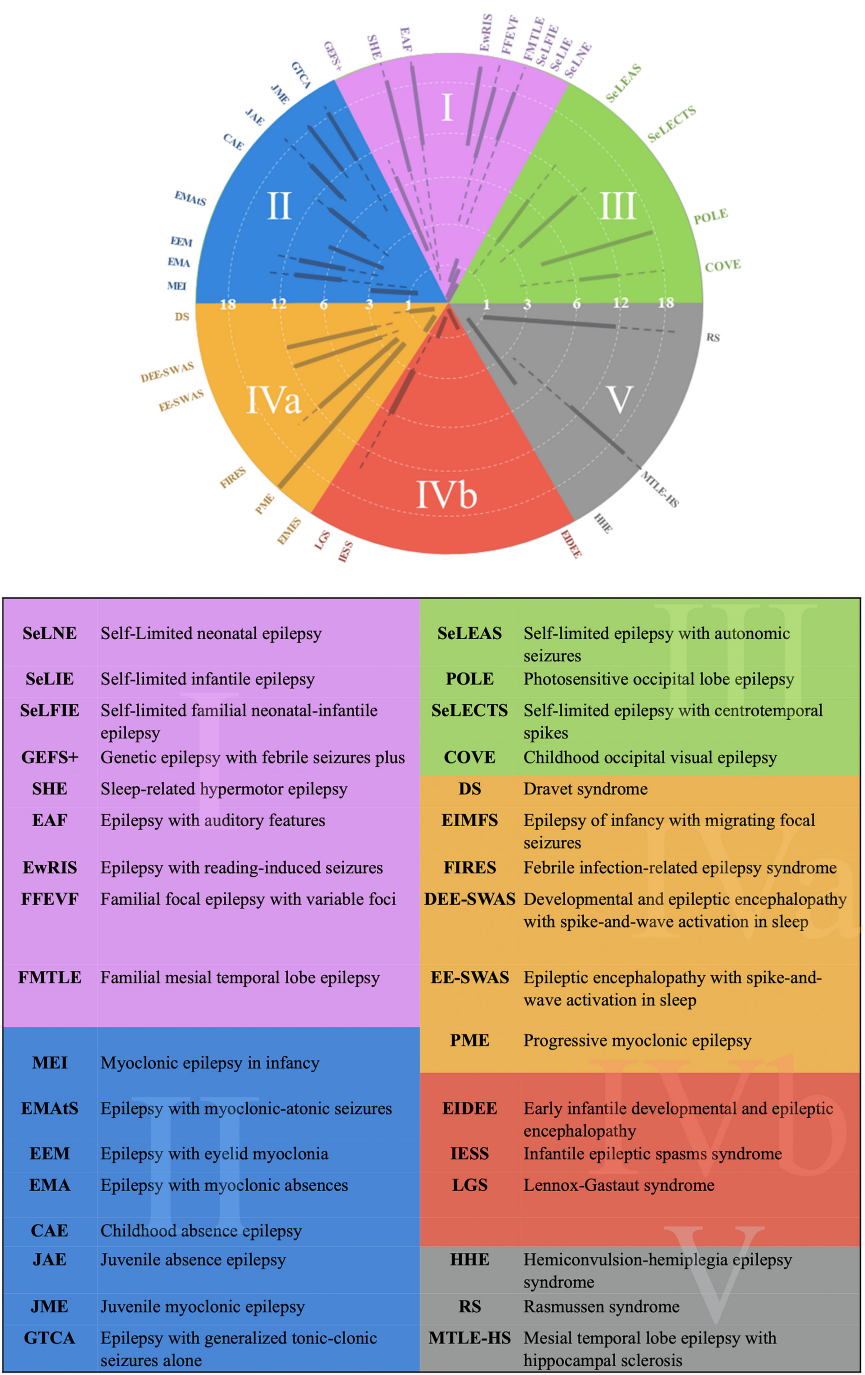
An EEG‐ based categorization of the epilepsies and legend.

We created a diagram to match the three‐dimensional figure developed by Nabbout et al., with a slightly different orientation to align with our EEG categories and show this as Figure 2. The numbering of the categories correlates with the chances of a positive family history, with type I EEGs (normal studies) being the most commonly seen pattern in epilepsies that are inherited in an autosomal dominant fashion.

To demonstrate how the one can use the figure consider we can walk through our typical method of teaching. First, we begin with the anamnesis, noting the presence of any factors that might increase the likelihood of epilepsy, the development of the child, the age of presentation, the semiology of the seizures and the results of the neurological examination. Next, we consider the EEG findings, where the EEG background is paramount. If the background recorded awake and asleep is normal, then the EEG category most likely lies in the northern hemisphere. (This should correlate with normal development of the child and a normal neurological examination). If there is a strong family history consistent with autosomal dominance inheritance, and the interictal EEG is normal (EEG Category I), then an AD or Familial epilepsy is suggested. If there are generalized spike–wave discharges superimposed on a normal background (EEG Category II), then a GGE is considered. (It is important to note that rhythmic slowing, such as FIRDA or OIRDA may be seen in the EEG background of these patients as a notable exception.) If the EEG shows focal discharges, and particularly those that are bilateral and independent then (EEG Category III) then an associated with self‐limited focal epilepsies is likely.

In contrast if the EEG background shows slowing, then the EEG category is within the southern hemisphere of the figure. If the slowing is generalized or diffuse, consistent with EEG Category IVa, then a DEE is the likely diagnosis. If the slowing is focal then EEG Category V is likely, and this is associated with a focal structural epilepsy.

A subset of EEGs with diffuse background slowing also demonstrate discontinuity, electrodecrements or both of the background (EEG Category 4b). In the diagram this category is juxtaposed to the focal structural category because there are important circumstances where focal structural lesions in children can cause epileptic encephalopathies.

Seizure semiology can be easily superimposed. Seizures associated with EEG category I may be either focal or apparently generalized, though the predominant seizure type is focal. Generalized seizures are the corresponding seizure type associated with EEG Category II. The seizures associated with EEG Category III are focal, often with alternating laterality. The seizure semiology in those epilepsies associated with EEG Category IVa tends to be pleomorphic or mixed. Prominent seizure types found in Category IVb are spasms and tonic seizures, or mixtures of the two. Finally, focal seizures predominate in patients whose EEGs show features consistent with Category V.

Accurately categorizing the EEG and using it to help guide diagnosis of an epilepsy syndrome in turn helps the treating neurologist better understand appropriate workup and treatment options. It may also help guide future research and triage for optimal patient care. Category IVa EEG's have been highly associated with pathogenic variants responsible for epilepsy. These epilepsy syndromes are often drug resistant and are likely to be best managed with a prompt referral to a tertiary care epilepsy center, where there may be access to innovative therapies including treatments that are gene modifying (e.g. Dravet Syndrome). Category IVb epilepsy syndromes may be better understood though advanced genetic workup such as whole exome sequencing/ whole genome sequencing versus epilepsy gene panel testing. These patients may benefit from a thorough evaluation by a geneticist or a neurologist that is very familiar with genetic evaluations. Patients with Category V EEG's may develop drug resistant epilepsy and be surgical candidates. Thus, consideration of technologies such as EEG source localization, and advanced imaging are important to consider in drug‐resistant epilepsies with Category V EEGs.

We believe that the figure may help the next generation of pediatric neurology trainees as they grapple with the step‐wise diagnosis of epilepsy syndromes. More importantly, we hope that the figure will ultimately help clinicians secure accurate diagnoses for their patients leading to prompt treatment and cessation of seizures as we continue in our communal quest to cure epilepsy.

## CONFLICT OF INTEREST statement

None of the authors have any conflict of interest to disclose. We confirm that we have read the Journal's position on issues involved in ethical publication and affirm that this report is consistent with those guidelines.
